# ACF Based Region Proposal Extraction for YOLOv3 Network Towards High-Performance Cyclist Detection in High Resolution Images

**DOI:** 10.3390/s19122671

**Published:** 2019-06-13

**Authors:** Chunsheng Liu, Yu Guo, Shuang Li, Faliang Chang

**Affiliations:** School of Control Science and Engineering, Shandong University, Ji’nan 250061, China; liuchunsheng@sdu.edu.cn (C.L.); yguo@mail.sdu.edu.cn (Y.G.); lskzxysdu@gmail.com (S.L.)

**Keywords:** cyclist detection, region proposal extraction, aggregated channel feature (ACF), You Only Look Once (YOLO)

## Abstract

You Only Look Once (YOLO) deep network can detect objects quickly with high precision and has been successfully applied in many detection problems. The main shortcoming of YOLO network is that YOLO network usually cannot achieve high precision when dealing with small-size object detection in high resolution images. To overcome this problem, we propose an effective region proposal extraction method for YOLO network to constitute an entire detection structure named ACF-PR-YOLO, and take the cyclist detection problem to show our methods. Instead of directly using the generated region proposals for classification or regression like most region proposal methods do, we generate large-size potential regions containing objects for the following deep network. The proposed ACF-PR-YOLO structure includes three main parts. Firstly, a region proposal extraction method based on aggregated channel feature (ACF) is proposed, called ACF based region proposal (ACF-PR) method. In ACF-PR, ACF is firstly utilized to fast extract candidates and then a bounding boxes merging and extending method is designed to merge the bounding boxes into correct region proposals for the following YOLO net. Secondly, we design suitable YOLO net for fine detection in the region proposals generated by ACF-PR. Lastly, we design a post-processing step, in which the results of YOLO net are mapped into the original image outputting the detection and localization results. Experiments performed on the Tsinghua-Daimler Cyclist Benchmark with high resolution images and complex scenes show that the proposed method outperforms the other tested representative detection methods in average precision, and that it outperforms YOLOv3 by 13.69% average precision and outperforms SSD by 25.27% average precision.

## 1. Introduction

In many countries, pedestrians and cyclists are the most vulnerable road users (VRUs) in traffic crashes. It is easier for cyclists to get involved in traffic crashes because of their relatively fast speed. In recent years, a lot of research focused on developing Advanced Driver Assistance Systems (ADAS) for anti-collision of VRUs [1,2]. The detection of VRUs including cyclists and pedestrians is still a difficult problem, due to the difficulties brought by diverse cyclist postures, small-size, occlusions and relative fast speed, etc.

Many technologies have been proposed in the past decades. The main technological approaches for detection can be divided into two major approaches: sensor-based detection methods and vision-based detection methods. Sensors include liDAR, radar, infrared sensor and so on. Vision-based detection methods have been proved to have the ability to solve complex tasks, such as face detection [3], traffic sign detection [4] and pedestrian detection [5], etc. With cheap price and easy installment, vision based sensor is a nature solution for detection.

Compared with cyclist detection, pedestrian detection has received much attention for decades. More than 40 pedestrian detection methods based on machine learning were analyzed in [6]. The AdaBoost and Cascade based detection structure [7] was applied in pedestrian detection in 2003. The milestone Histogram of Oriented Gradients (HOG) and Support Vector Machine (SVM) based structure [8] was proposed to address pedestrian detection problem. The Deformable Part Model (DPM) was designed for pedestrian detection in [9]. The ChnFtrs with multiple registered image channels was proposed for detection in [10]. In 2014, locally decorrelated channel feature (LDCF) was utilized for pedestrian detection [5]. In addition, Cao et al. [11] proposed two types of non-neighboring features and applied them to detecting pedestrians. Du et al. [12] applied their Fused DNN detector to pedestrian detection. Zhang et al. [13] analyzed the performance of Faster R-CNN in pedestrian detection and made improvements. Paisitkriangkrai et al. [14] introduced a new structured ensemble learning approach and extracted low-level features based on spatial pooling for pedestrian detection. Tian et al. [15] designed DeepParts for handling occlusion problem in pedestrian detection. Although many methods have been proposed, pedestrian detection is still a challenging task [16]. Compared with pedestrian detection, cyclist detection is a more difficult problem due to different postures, different riding tools, and blur caused by fast speed, etc. Additionally, because of the relatively fast speed, it is easier for cyclists to get involved in traffic crashes.

In the recent decade, many cyclist detection methods were proposed. In 2010, Li et al. [17] designed a detection method that combines local features, global features and linear SVM classifier. In [18], a two-stage multi-model bicyclist detection method was proposed based on region of interest (ROI) detection and integral features. In [19], viewpoint-based detector with cascaded HOG features were built for cyclist detection. Cho et al. [20] introduced multiple patch-based Lucas-Kanade tracker to HOG and SVM based detector for cyclist detection in consecutive frames. In 2017, Li et al. [21] presented a detection proposal method for cyclist detection including a cyclist shared salient region detection part, a redundancy and geometric constraint based detection part, and a deep convolutional neural network. In the same year, Li et al. [22] presented a unified framework for concurrent pedestrian and cyclist detection, which includes an upper body detector, a discriminative deep model based on Fast R-CNN and a postprocessing step.

Cyclist detection belongs to the object detection problem. In recent decades, some powerful features, such as Haar [23] and HOG [8], were proposed to express objects. Combined with powerful features, some efficient detection frameworks, such as cascade [23] and fast pyramid [24], were proposed to improve the detection performance. Deep learning have made breakthroughs in detection in recent years. Many algorithms, such as Region Convolution Neural Networks (R-CNN) [25], Fast R-CNN [26], Faster R-CNN [27], Single Shot MultiBox Detector (SSD) [28] and You Only Look Once (YOLO) [29], were proposed to address different object detection problems.

As one of the most representative networks, YOLO network can get good performance in both speed and accuracy and has high potential in the cyclist detection problem. Shortly after the advent of YOLO, the author of YOLO has improved the network. In 2016, YOLOv2 [30] was presented. Furthermore, in 2018, YOLOv3 [31] was presented. YOLOv3 has higher accuracy than the previous version and it is still fast. YOLO has good performance on some datasets, such as PASCAL VOC [32], KITTI dataset [33], COCO [34] and ImageNet [35]. Furthermore, in recent years, YOLO has been applied in many fields, such as pedestrian detection [36], license plate detection [37], vehicle detection [38], and traffic sign detection [39], etc. Therefore, the YOLO network has very high potential in the cyclist detection problem.

However, though some deep learning based networks such as YOLO and SSD can detect objects in some detection areas quickly and accurately, these methods usually cannot achieve good performance when dealing with the cyclist detection problem. The main reason is that these methods cannot perform well on detecting small-size objects in high resolution images.

We assume that if the potential regions can be gotten first, the high-resolution images can be cropped into some regions of interest (ROI). The YOLO or SSD based methods can be performed on these small regions to achieve better performance. Following this hypothesis, we design a cyclist detection framework based on YOLO network. The proposed framework has three main parts.

Firstly, in order to extract region proposals from high resolution images, based on aggregated channel feature (ACF) [40], we propose a region proposal extraction method called ACF based region proposal (ACF-PR) method. In ACF-PR, we firstly design an ACF based detector to fast extract candidates, and then a bounding boxes merging and extending method is designed to merge the bounding boxes into correct region proposals for the following YOLO net. Then, a suitable YOLO net is designed for fine cyclist detection in the region proposals generated by ACF-PR. Lastly, we design a post-processing step, in which the results of YOLO net are mapped into the original image outputting the detection and localization results. The proposed cyclist detection structure is evaluated on the public Tsinghua Daimler Cyclist Benchmark (TDCB) [41] and it outperforms the other representative methods in our comparison.

The paper is organized as follows. In Section 2, the proposed cyclist detection structure including a novel region proposal method, a deep learning network and a specific post-processing step is presented. Section 3 shows the evaluation results and comparison with other representative detection methods, and Section 4 gives the final conclusion and future work.

## 2. Proposed Methods

Images taken by the on-board camera for cyclist detection are usually with high resolution. However, dealing with high resolution images, the general deep learning algorithms including YOLO, SSD and Faster-RCNN have relatively poor performance. During experiment, we found that if the high-resolution image can be cropped into small regions that contain objects, some deep learning based networks can perform well on these small regions.

**Figure 1:**
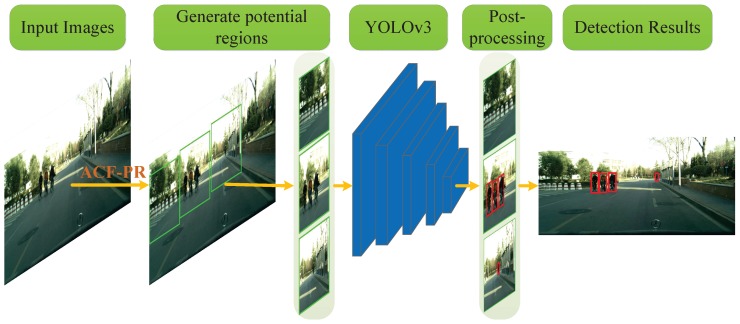
Overview of the proposed cyclist detection method. Color images are first processed by the aggregated channel feature region proposal (ACF-PR) method. The proposed ACF-PR method utilizes ACF to get region candidates, and then it performs the analysis of these candidates to generate potential regions. Then the YOLO network utilizes these potential regions as inputs to do fine detection and localization. At last, to get the final result, a post-processing step is performed to merge and map the bounding boxes.

Following this methodology, a novel cyclist detection structure is proposed, which contains three main parts. (1) ACF based region proposal (ACF-PR) method, (2) YOLO based cyclist detection method, and (3) a post-processing step for fine localization. The overall architecture of the proposed cyclist detection structure is shown in Figure 1.

Firstly, the ACF-PR region proposal extraction method is designed. An ACF detector is trained to detect coarse ROIs containing cyclists. Because the regions generated by ACF usually just contain parts of cyclists, we design boxes merging and extending method to merge the bounding boxes into correct region proposals for the following YOLO net. Then, a suitable YOLOv3 net is utilized for fine detection of cyclists in the region proposals generated by ACF-PR. Lastly, we design a post-processing step, in which the results of YOLO net are selected and mapped into the original image resulting in the detection and localization results.

### 2.1. ACF-PR Region Proposal Generating Method

**Figure 2:**
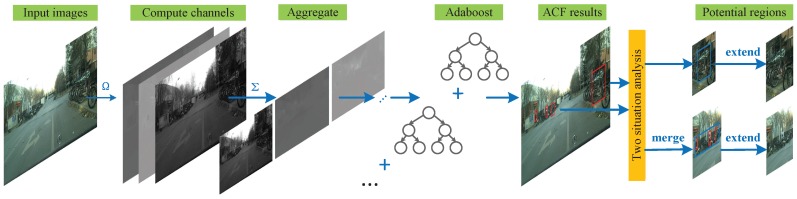
Structure for ACF-PR region proposal method. To get potential regions, ACF detector first gets preliminary bounding boxes. Then these bounding boxes are divided into two cases according to their position for different processes; lastly, potential regions are generated.

The original ACF method has achieved good performance in some detection problems. In this paper, we explore the novel use of ACF for region proposal extraction. If ACF is directly performed for cyclist detection, the detected regions may contain just part of the cyclists and many false positives. To resolve this problem, we propose the ACF-PR region proposal generating method. In this method, instead of directly using the generated region proposals for classification like most region proposal methods do, we generate large potential regions containing objects for the following deep network. Using this methodology, regions containing only part cyclists can be avoided. The structure of ACF-PR is shown in Figure 2.

Given an input image, the ACF computes several channels, sums every block of pixels, smooths the resulting lower resolution channels and uses boosting to distinguish objects. ACF builds a fast feature pyramid $P=\{p_{1},p_{2},\ldots ,p_{n}\}$, here *n* represents the number of layers. The channels used are the same as [40]: normalized gradient magnitude (1 channel), histogram of oriented gradients (6 channels), and LUV color channels (3 channels), for a total of 10 channels.

The boosting is an integrated learning algorithm that linearly combines weak classifiers into a strong classifier,

(1) $$\begin{array}{c} F\left(x\right)=sign(\sum_{i=1}^{N}a_{m}G_{m}\left(x\right)), \end{array}$$

where, $G_{m}$ represents a weak classifier and $a_{m}$ is the weight of $G_{m}$ in strong classifier. sign(x) is the symbolic function. When training, the classifier produced in the next iteration was trained on the basis of the previous iteration,

(2) $$\begin{array}{c} F_{m}\left(x\right)=F_{m-1}\left(x\right)+a_{m}G_{m}\left(x\right), \end{array}$$

where $F_{m}\left(x\right)$ represents the classifier produced in the *m*th iteration.

The loss function is,

(3) $$\begin{array}{c} L(Y,f(x))=exp(-Yf(x)), \end{array}$$

where *Y* represents the label of *x*, the f(x) represents the result we generate. We determine $a_{m}$ according to the minimum principle of the loss function L(Y,f(x)).

In this paper, Adaboost is used to train and combine 4096 2-depth decision trees over the h/4·w/4·10 aggregated features, where h×w is the input window and 4 is the down sample scale. Based on these parameters, we can get the best performance in our experiments. In the detection process, multi-scale sliding-window is used to scan the image and generate aggregate channel feature, and these features are sent into Adaboost.

To adapt to the cyclist sizes in this study, *modelDs* (model height and width without padding) is set to (50,32) and *modelDsPad* (model height and width with padding) is set to (64,48). The *nNeg* (max number of negative windows to sample) is set to 10,000 and the *nAccNeg* (max number of negative windows to accumulate) is set to 30,000.

Using these training processes, we train an ACF detector to perform preliminary detection. Instead of arranging ACF bounding boxes according to the level of confidence like traditional ACF does, the bounding boxes in this method are reordered from left to right and from top to bottom in the image.

In detection, one cyclist detected by ACF may have several different bounding boxes, which causes many false positives in the detection process. During experiments, we found that the distances between the bounding boxes belonging to one cyclist are not far away. We design a merging method for merging bounding boxes belonging to the same object. In this process, all bounding boxes are divided into two cases according to the distances between the bounding boxes. In one case, two bounding boxes are partially overlapped or the distance is short. In the other case, two bounding boxes are far away from each other.

**Figure 3:**
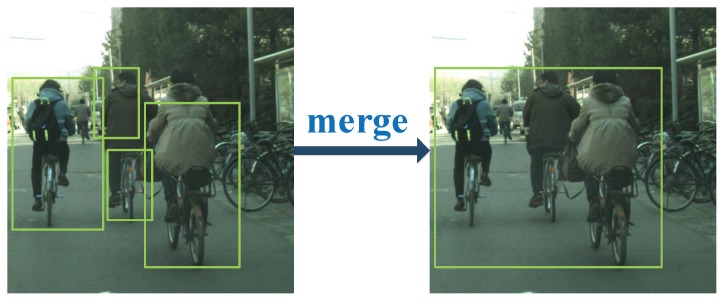
One example of the process of merging bounding boxes.

- In one case, each detected cyclist instance is marked with several different bounding boxes. In order to merge bounding boxes into a correct one and get the entire cyclist instance, two small boxes are merged into one when the distance between them is within a certain range. To show the merging process intuitively, an example for merging is provided in Figure 3. We use $x_{min}$, $y_{min}$ to represent the minimum value of the *x*, *y* coordinate on two boxes. Then,
  (4) $$x_{min}=\left\{\begin{array}{cc} x_{b1}, & x_{b1}<x_{b2},\\ x_{b2}, & x_{b1}\geq x_{b2}, \end{array}\right.$$
  (5) $$y_{min}=\left\{\begin{array}{cc} y_{b1}, & y_{b1}<y_{b2},\\ y_{b2}, & y_{b1}\geq y_{b2}, \end{array}\right.$$
  where, $\left(x_{b1},y_{b1}\right)$ and $\left(x_{b2},y_{b2}\right)$ are the coordinates of the top-left point of two bounding boxes. We use $x_{t}$ and $y_{t}$ to represent the maximum distance threshold of two bounding boxes which can be merged on *x*, *y* coordinates. The value of $x_{t}$ and $y_{t}$ should ensure that the potential regions eliminate the situation where only half of the object is contained. The values of $x_{t}$ and $y_{t}$ are fixed to ensure that the potential regions contain the whole objects; hence, both $x_{t}$ and $y_{t}$ are set to 832 that is the maximum size of the cyclist instances. $\left(w_{b1},h_{b1}\right)$ and $\left(w_{b2},h_{b2}\right)$ represent the width and height of two bounding boxes. If $x_{min}+x_{t}$ and $y_{min}+y_{t}$ satisfy the condition,
  (6) $$\left\{\begin{array}{c} x_{min}+x_{t}\geq x_{b1}+w_{b1},\\ x_{min}+x_{t}\geq x_{b2}+w_{b2}, \end{array}\right.$$
  (7) $$\left\{\begin{array}{c} y_{min}+y_{t}\geq y_{b1}+h_{b1},\\ y_{min}+y_{t}\geq y_{b2}+h_{b2}, \end{array}\right.$$
  then, we can get one large bounding box by merging two bounding boxes. Comparing $x_{b1}+w_{b1}$ and $x_{b2}+w_{b2}$ to get the maximum *x* coordinate of two bounding boxes. Based on this maximum value, we can calculate the width of merged bounding box. Similarly, $y_{b1}+h_{b1}$ and $y_{b2}+h_{b2}$ are discriminated and used to calculate the height of merged bounding box.
  (8) $$\begin{array}{c} x_{b}=x_{min}, \end{array}$$
  (9) $$\begin{array}{c} y_{b}=y_{min}, \end{array}$$
  (10) $$w_{b}=\left\{\begin{array}{cc} x_{b1}+w_{b1}-x_{min}, & x_{b1}+w_{b1}>x_{b2}+w_{b2},\\ x_{b2}+w_{b2}-x_{min}, & x_{b1}+w_{b1}\leq x_{b2}+w_{b2}, \end{array}\right.$$
  (11) $$h_{b}=\left\{\begin{array}{cc} y_{b1}+h_{b1}-y_{min}, & y_{b1}+h_{b1}>y_{b2}+h_{b2},\\ y_{b2}+h_{b2}-y_{min}, & y_{b1}+h_{b1}\leq y_{b2}+h_{b2}, \end{array}\right.$$
  where, $\left(x_{b},y_{b}\right)$ represents the top-left point of the merged bounding box and $\left(w_{b},h_{b}\right)$ represents the width and height of the merged bounding boxes. After merging two bounding boxes into one large bounding box that may contain one object or several objects. These merged bounding boxes are extended as potential regions and as inputs for the following network.
- In the other case, two bounding boxes are far apart from each other, which means that these boxes are for different instances and do not need to merge. In this case, the bounding box may contain the entire object instance, and sometimes also may contain part of the object instance or just background. For fine detection and localization, these bounding boxes also need to be sent into the following deep network for further detection. If the distance between two bounding boxes is not within a certain range, these boxes are regarded as two separate objects. In order to contain as many entire object instances as possible, these bounding boxes are extended as potential regions and as inputs for the following network.

Bounding boxes are all extended to m×m pixels to be served as potential regions, which ensures that the potential regions contain the whole objects. In this study, *m* is set to 832 that is the maximum size of the cyclist instances. We crop the potential regions according to the coordinates. The relationship between the potential region and the bounding box is,

(12) $$\begin{array}{cc} 2(x_{po}-x_{b}) & =w_{b}-m,\\ 2(y_{po}-y_{b}) & =h_{b}-m, \end{array}$$

where, $\left(x_{po},y_{po}\right)$ indicate the (x,y) coordinates of the top-left point of the potential region in the original image; $\left(x_{b},y_{b}\right)$ indicate the (x,y) coordinates of the top-left point of the bounding box before extending; $w_{b}$ and $h_{b}$ indicate the width and height of the bounding box. At last, these cropped potential regions are sent into the subsequent network.

**Figure 4:**
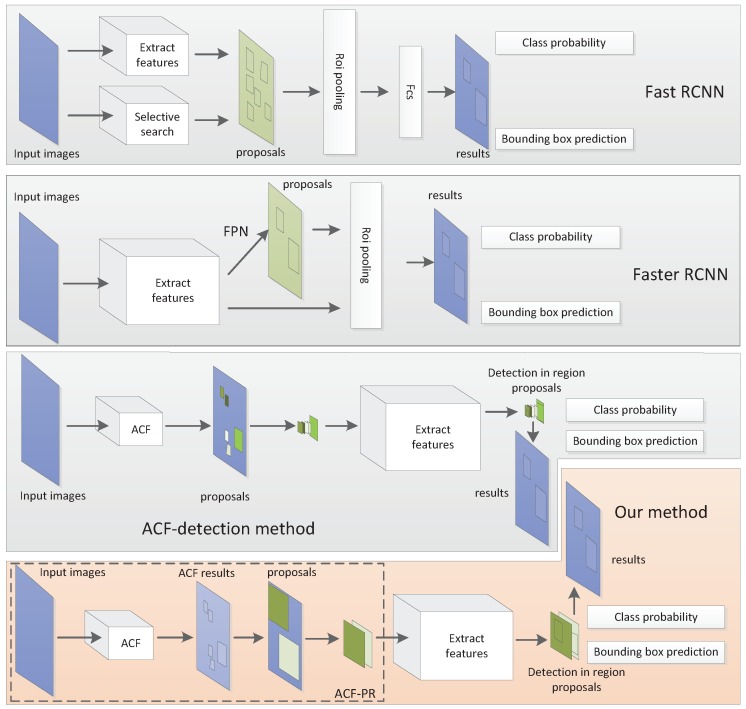
The structure of our method and other methods.

To illustrate the advantage of ACF-PR, we compare the structures of Fast R-CNN [26], Faster R-CNN [27], ACF-detection method and our method in Figure 4. Fast R-CNN uses selective search (SS) method to generate bounding boxes. Experiments in [22] show that SS-FRCN (Fast R-CNN with selective search method) needs about 23 s per image (2048×1024) when detecting. It is too slow for detection. Hence, SS is not suitable for region proposals extraction in cyclist detection.

Region proposal methods like region proposal network (RPN) in Faster R-CNN generate and select bounding boxes directly, and these bounding boxes are used for regression and classification. With high resolution input images (2048×1024) and a large range of object sizes from 20 pixels to 800 pixels, the feature map of convolutional network may lose some details and make it difficult to detect small size objects. Hence, RPN is not suitable for detecting small-size objects in high resolution images.

Different from RPN, our method only generates potential regions in which cyclists may appear; then these potential regions are sent into the following network for detection. We extract features from these potential regions rather than extracting features to generate potential regions. The main function of ACF-PR is to lessen the detection range. We do not utilize ACF as a region proposal method directly, because that ACF-detection method shown in Figure 4 usually generates bounding boxes with less than half of the instance. The experiments show that only approximately 69% cyclists are contained in the detection results of ACF. If these bounding boxes are sent into detection network, the detection rate will not be higher than 69%. However, our proposed ACF-PR method can contain 100% cyclists, which ensures the relatively good detection result. In addition, it only takes about 0.18 s per image (2048×1024) with Central processing unit (CPU) to generate potential regions, which ensures the relatively fast speed of detection.

Hence, our proposed ACF-PR method is more suitable for cyclist detection than the other region proposal methods, when dealing with images with high resolution.

### 2.2. YOLO Network for Cyclist Detection

YOLO has proved to have the ability to handle complex tasks, such as pedestrian detection [36], license plate detection [37], vehicle detection [38], and traffic sign detection [39], etc. YOLO is a single deep network which can get predicted bounding boxes and class probabilities at the same time, achieving high accuracy and extremely fast speed.

**Figure 5:**
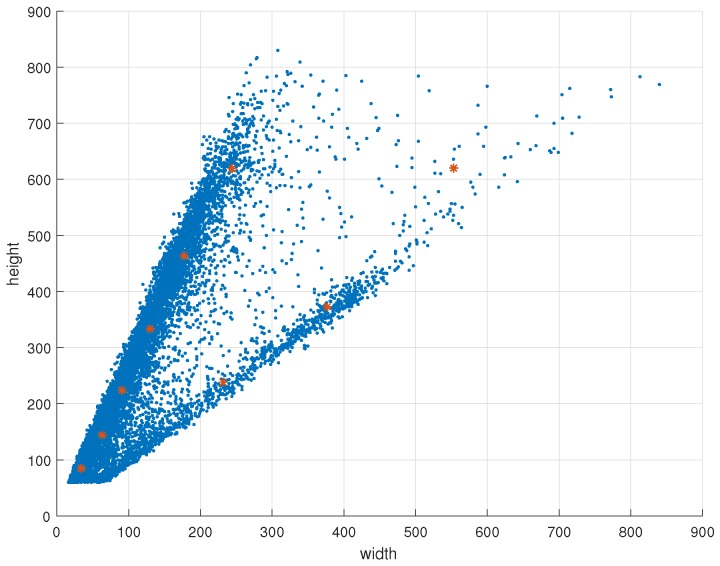
The K-means clustering result of training set. The x-axis and y-axis represent the width and height of bounding boxes in pixels. The blue point represents the instance and the red point represents the clustering point.

**Figure 6:**
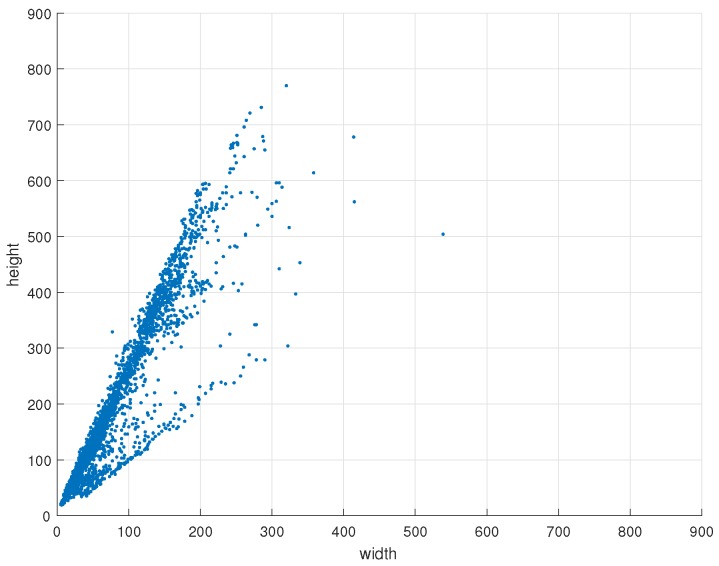
The distribution of cyclist instances in test set. The x-axis and y-axis represent the width and height of the ground-truth in pixels. One blue point represents one cyclist instance.

In this study, we designed a suitable detector based on YOLOv3 net for fine detection and localization. YOLOv3 has 106 layers, including successive 3×3 and 1×1 convolutional layers, shortcut connections, up-sample layers, route layers and detection layers. Figure 5 and Figure 6 show that almost all of the sizes of cyclists in this study are less than 832, so the input size of YOLOv3 is set to 832×832. The structure is shown in Table 1. Shortcut connections have similar construction with ResNet [42]. The route layers are to combine two feature maps or get the feature map of a previous layer. The function of the up-sample layer is to up-sample the feature map with a stride of 2 via bilinear interpolation. In addition, batch normalization layer [43] is utilized to make improvements in convergence. We do not list this layer in Table 1, because each convolutional layer is followed by a batch normalization layer.

Unlike the previous version, YOLOv3 predicts boxes at three different scales. From Table 1, the three detection layers are designed to preform prediction at three different scales. The similar concept of feature pyramid networks [44] is used to extract features from these three scales. It means that YOLOv3 divides the input image into three different sizes of grid: $S_{1}\times S_{1}$, $S_{2}\times S_{2}$, $S_{3}\times S_{3}$. If the center of an object is in a grid cell receptive field, this grid cell is responsible for detecting this object. Each grid cell predicts three bounding boxes. Thus, the number of YOLOv3 anchors is 9 and the number of bounding boxes it can get is $(S_{1}\times S_{1}+S_{2}\times S_{2}+S_{3}\times S_{3})*3$. These bounding boxes are analyzed and selected to get final detection results. Comparing with the previous version, YOLOv3 can get much better detection performance and the speed of it is still fast.

In order to get the anchors that YOLOv3 needs, K-means clustering is utilized to determine bounding box priors. We set our anchors on the clustering result of K-means. In this network, the number of anchors is set to 9, which is the same as [31]. At each scale, each cell uses three anchors to predict three bounding boxes.

The distribution of cyclist instances in the training set and test set are shown in Figure 5 and Figure 6 respectively. The coordinate *x* and *y* indicate the width and height of the ground-truth. Each blue point indicates one instance. Comparing the data in Figure 5 and Figure 6, the distributions of these two sets are similar. We use K-means method to get nine clusters and set anchors according to the results of K-means. The result is shown in Figure 5, each red point indicates one clustering point. In this work, the nine clusters are: (33, 84), (62, 143), (93, 221), (129, 333), (177, 464), (244, 620), (252, 258), (384, 378), (557, 623).

The inputs of YOLOv3 we used are outputs of ACF-PR. Therefore, the size of potential regions *m* is set to 832, which is equal to the size of input size of YOLOv3. The outputs of YOLOv3 are inputs of the post-processing process.

### 2.3. Post-Processing

The detection results of YOLOv3 are based on potential regions, and need to be mapped into the original image. Because some potential regions may be partially overlapped when potential regions are generated, several bounding boxes may be generated for one same object. In order to solve this problem, a post-processing process including boxes mapping and non-maximum suppressing is designed. The coordinates of bounding boxes from YOLOv3 are based on potential regions and potential regions are gotten from original images. To get final detection results, the bounding boxes should be mapped from potential regions into original images. The relationship between coordinates of bounding boxes in potential regions and final coordinates in the original images is,

(13) $$\begin{array}{cc} x_{b} & =x_{bp}+x_{po},\\ y_{b} & =y_{bp}+y_{po}, \end{array}$$

where, (x,y) indicate the *x* and *y* coordinates; the subscript *b* is for the bounding boxes in the original image; the subscript bp is for the bounding boxes in the potential regions; the subscript po is for the potential regions in the original image. The width, height and class of bounding boxes do not change when they are mapped into the original image.

After mapping, overlapping detections may appear. One cyclist instance may have a number of bounding boxes associated with it. Non-maximum suppression (NMS) is utilized to eliminate repeated detections. b1 and b2 represent two bounding boxes. *t* represents the threshold. IoU(b1,b2) is defined,

(14) $$\begin{array}{c} IoU\left(b1,b2\right)=\frac{b1\cap b2}{b1\cup b2}, \end{array}$$

where, b1∩b2 means the area of overlap, b1∪b2 means the area of union. If IoU(b1,b2)>t, the NMS step retains the bounding box with the highest score as the detection result. After this process, the detection results of YOLOv3 are mapped into the original image.

## 3. Experiments

### 3.1. Dataset and Evaluation Protocol

Comparing with pedestrian detection, cyclist detection receives far less research attention. The public challenging cyclist datasets is rare. Before 2016, only the KITTI object detection benchmark has cyclist instances; however, the number of cyclist instances is less than 2000, which is insufficient for cyclist training and testing. In 2016, the public Tsinghua-Daimler Cyclist Benchmark (TDCB) [41] was proposed, which contained more than 10,000 annotated cyclists.

As the only public cyclist detection dataset, TDCB still has some problems. Firstly, some cyclists in this dataset are invisible even to human eyes because of high similarity with background, small-size or occlusions. Secondly, some small cyclists cannot be distinguished from small motorcyclists. Thirdly, the samples in its training set were captured under similar weather and light conditions, and cannot cover very different weather and light conditions in the test set. These three problems may result in bad generalization ability. Hence, in our experiments, we rebuilt the training set, the validation set and the test set.

We firstly merge the original test and validation sets to a merged set. The 2758 images in the merged set are randomly selected to form the new test set. The new rebuilt training set has 10,000 images, including 380 images randomly selected from the merged set and 9620 images in the original training set; the rest of the images in the merged set form the new validation set. In this way, the weather and light conditions in these three rebuilt sets do not show many differences. After sets rebuilding, the percentages of the new training set, test set and validation set are approximately 70%, 20% and 10% respectively.

The method used in the PASCAL object challengers [32] is utilized here to show the relationship between precision and recall rate. Here, we use *P* to represent precision and *R* to represent recall. The precision and recall are calculated as,

(15) $$P=\frac{TP}{TP+FP},$$

and,

(16) $$R=\frac{TP}{TP+FN},$$

where, TP indicates the number of true positives, FP indicates the number of false positives and FN indicates the number of false negatives.

**Figure 7:**
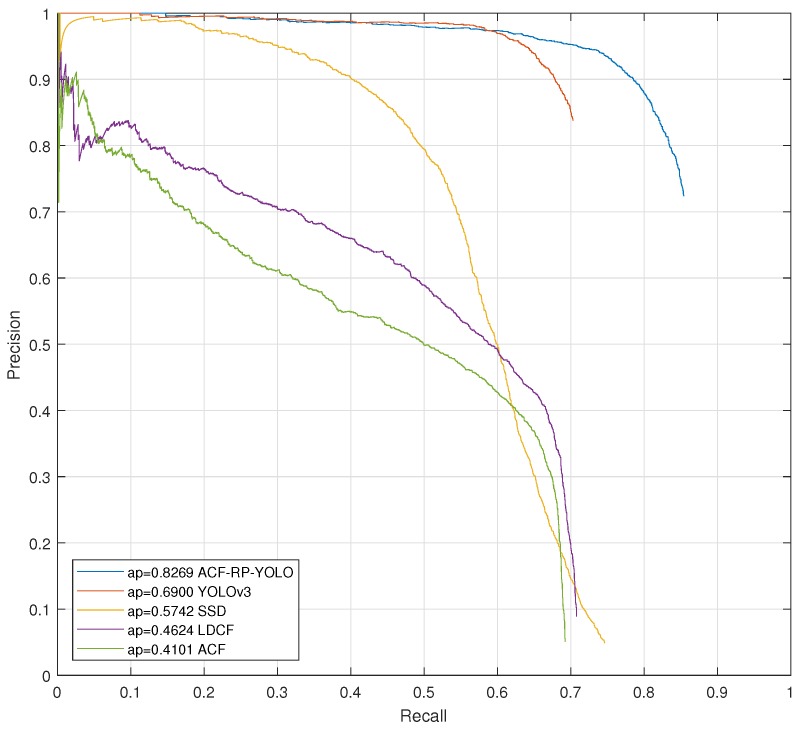
Precision versus recall curves of various detectors with different settings in Tsinghua-Daimler Cyclist Dataset. The average precision (AP) is listed before the name of each method. ACF-PR-YOLO is the proposed method.

The average precision (AP) is used here to represent the performance of detector. AP is defined as,

(17) $$AP=\int_{0}^{1}P\left(R\right)dR,$$

where, *R* represents recall and *P* represents precision, both of which are between 0 and 1. P(R) here represents the curve composed of *P* and *R*. Figure 7 is an example. A larger value of AP means better performance. The PASCAL measure is used to assign the detection results to ground-truth objects, which means that the area of IoU overlap must exceed the threshold of 0.5. IoU is used here to measure the accuracy of detecting a corresponding object. IoU is defined as,

(18) $$IoU=\frac{DR\cap GT}{DR\cup GT},$$

where, DR represents the detection region, GT represents the ground-truth region. DR∩GT means the area of overlap, DR∪GT means the area of union. The threshold of IoU is set to 0.5, which means that if IoU is larger than 0.5, this object is considered as a successful detection.

Our proposed region proposal method of ACF-PR was designed based on the latest version of Dollár’s Computer Vision MATLAB Toolbox [40]. When training the detector, the cyclist instances were extracted from training set with bounding boxes higher than 60 pixels and fully visible, and the negative samples were from non-VRU set. YOLOv3 is open source. We trained our network based on the pre-trained model on ImageNet [35]. Batch size is 64. The value of max-batches is set to 206,000, and we used a learning rate of 0.0001 for 95,000 batches, and 0.00001 for the next 111,000 batches.

### 3.2. Evaluation of the ACF-PR Method

In this experiment, we compare ACF-PR with traditional ACF to show the efficiency and improvement of the proposed ACF-PR method.

ACF-PR is designed to generate potential regions in high resolution images. The potential regions are expected to contain all cyclist instances. The more cyclist instances in the potential area, the better the detection results can be. In this experiment, a cyclist instance with more than 50% area in the extracted potential regions is considered a region containing cyclist instances.

Table 2 shows that ACF-PR can generate potential regions that contain 100% cyclist instances while ACF can only contain 69.22% cyclist instances. The result means that the potential regions extracted from our ACF-PR contain 100% cyclist instances, which ensures the relatively good detection performance of the following detection process. The detection rate of 69.22% gotten from ACF means that the following detection rate will only be equal with or lower than 69.22%. In this study, 832×832 size potential regions are sent to YOLOv3 network for computing localization and classification; in this case, the average ratio of the area of all potential regions to the area of original images is 78%. The main reason why ACF-PR has such a large advantage is that it has the mechanism of boxes merging and extending, which largely reduces the cyclist instances that are half detected or missed.

### 3.3. Comparisons with Other Detection Methods

To evaluate the effectiveness of the proposed method, we compare the performance of our proposed method ACF-PR-YOLO with some other methods including YOLOv3 [31], SSD [28], LDCF [5] and ACF [40]. ACF-PR-YOLO represents the proposed method which utilizes ACF-RP for region proposal and YOLO for detection. YOLOv3 and SSD are two representative one-stage deep learning based detection methods. For YOLOv3, the class number is 1 and the other parameters are the same as [31]. The bone net of SSD we used was VGG16 and the input size of it was 300×300. LDCF and ACF are popular traditional detection methods. Due to the limited computer memory problem, we use 3798 images when training ACF and LDCF detectors. Though Li et al. has used TDCB dataset for cyclist detection, we did not compare with them because there was an additional large training set for training in [22] which is not publicly available.

The curves in Figure 7 show the overall detection performance of all detectors tested on the TDCB dataset. From Figure 7, we can find that ACF-PR-YOLO outperforms YOLOv3 by 13.69% AP and outperforms SSD by 25.27% AP. These results mean that the representative YOLOv3 and SSD nets have poor performance in the cyclist detection problem; the main reason is that the YOLOv3 and SSD nets have relatively poor performance in detecting small-size objects in high-resolution images. The proposed ACF-PR-YOLO outperforms by at least 13.69% better AP than these two methods. From the comparison of ACF-PR-YOLO and YOLOv3, it can also be concluded that the ACF-PR-YOLO has better AP than that of YOLOv3, because of using the proposed ACF-PR to generate proposal regions.

Figure 7 also illustrates that the proposed ACF-RP-YOLO method outperforms LDCF and ACF by 36.45% and 41.68% AP respectively. From experiments, we found that the LDCF and ACF methods usually extract just part of the objects or extract inaccurate object regions, which is the main reason for resulting in poor performance. This is also the reason why we use ACF to extract region proposals instead of directly using ACF for detection.

From the data in Figure 7, it can be concluded that the proposed ACF-PR-YOLO outperforms other methods in comparison. This achievement has three main reasons. Firstly, the ACF-PR can generate potential regions that contain 100% cyclists in the region proposal extraction process; this process can segment a high-resolution image into small potential regions, which can effectively avoid the poor performance of YOLOv3 on high-resolution images. Secondly, on the segmented small-size images, the YOLO can do fine detection achieving high performance on average precision. Thirdly, the designed post-processing method is designed to select the most suitable bounding boxes and to map them into a correct one.

Table 3 lists the detailed comparison with other popular detection methods including YOLOv3 [31], SSD [28], LDCF [5] and ACF [40], using parameters of AP, code type and consuming time per frame. In Table 3, the popular methods achieve relative low APs ranging from 41.01% to 69.00% in cyclist detection, while the proposed ACF-PR-YOLO can detect cyclists in a high AP of 82.69% and with an average consuming time of 0.35 s. Hence, the proposed ACF-PR-YOLO method can detect cyclists with high precision of 82.69% AP and small consuming time of 0.35 s. Our proposed method, running on a 3.20-GHz i5 CPU processor and a TITAN X GPU processor, needs about 0.35 s per image (2048×1024). The time consumption of these three parts is listed in Table 4. ACF-PR is written via Matlab and runs on a 3.20-GHz i5 CPU. It costs about 0.18 s per image. YOLO is written via C and it runs on a TITAN X GPU. The time it costs is about 0.164 s. The post-processing step is written in Matlab and runs in CPU. It costs 0.003 s.

**Figure 8:**
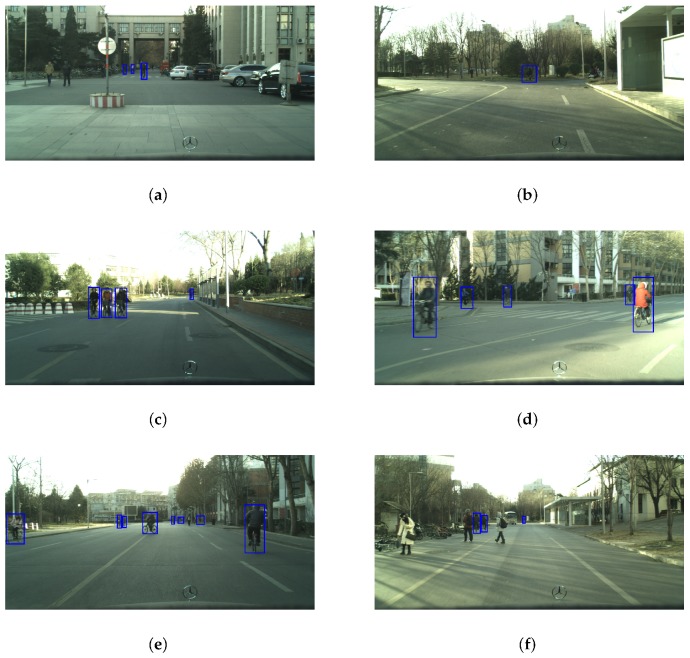

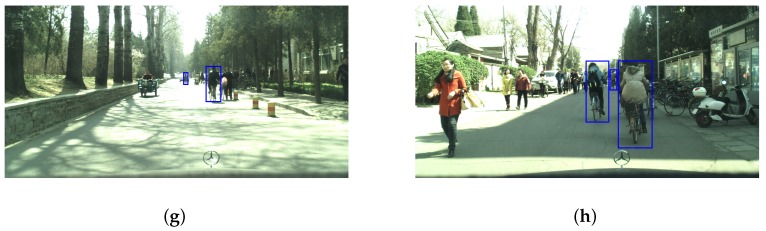
Some results of performing our detector on “Tsinghua-Daimler Cyclist Benchmark” with different scenarios. The images from (**a**) to (**h**) are the detection results in complex environments. In order to have a better visual effect, the contrast and brightness of the images above are enhanced for display. Blue bounding boxes represent detected cyclists.

Some results of performing our detector on the TDCB dataset with different scenarios are shown in Figure 8. Figure 8 illustrates that our detector can have good performance in different scenarios. Our method can not only detect cyclists in complex backgrounds and dense crowds, but also can separate cyclists from pedestrians.

## 4. Conclusions

Some representative fast deep networks including YOLO and SSD usually cannot achieve high precision when dealing with small-size objects and high resolution images. To overcome this problem, a framework for cyclist detection in large high-resolution images is presented in this paper. The framework contains an ACF-PR region proposal method, a YOLOv3 net for cyclist detection and a post-processing step.

In order to extract potential regions from high resolution images, the region proposal method of ACF-PR is proposed. In ACF-PR, an ACF detector is firstly utilized to fast extract candidates; then a bounding boxes merging and extending method is designed to merge the bounding boxes into correct region proposals for the following YOLO net. Then a suitable YOLOv3 net is designed to do detection in the potential regions generated by ACF-PR. The YOLOv3 net has a better performance on small-size potential regions rather than that on high-resolution original images. Lastly, a post-processing step is performed to select the most suitable bounding box and to map it into original images with high resolution. We evaluate our method on the public TDCB dataset and compare it with other representative methods. The experiments demonstrate that it outperforms the representative methods in our comparison, and that it outperforms YOLOv3 by 13.69% average precision and outperforms SSD by 25.27% average precision.

Although our algorithm is designed for cyclist detection, it has great potential for other object detection. In the future, in order to improve detection performance, we plan to develop an efficient detection algorithm that can adapt to more complex scenarios. Instead of designing a single frame detector, we plan to do detection based on video and do research on the feature relationship between consecutive video frames.

## Figures and Tables

**Table 1:** The structure of the YOLOv3 net.

	Type	Filters	Size	Output
	conv	32	3×3	832×832
	conv	64	3×3/2	416×416
	conv	32	1×1	416×416
1×	conv	64	3×3	416×416
	shortcut			416×416
	conv	128	3×3/2	208×208
	conv	64	1×1	208×208
2×	conv	128	3×3	208×208
	shortcut			208×208
	conv	256	3×3/2	104×104
	conv	128	1×1	104×104
8×	conv	256	3×3	104×104
	shortcut			104×104
	conv	512	3×3/2	52×52
	conv	256	1×1	52×52
8×	conv	512	3×3	52×52
	shortcut			52×52
	conv	1024	3×3/2	26×26
	conv	512	1×1	26×26
4×	conv	1024	3×3	26×26
	shortcut			26×26
3×	conv	512	1×1	26×26
	conv	1024	3×3	26×26
	conv	18	1×1	26×26
	detection			
	route			
	conv	256	1×1	26×26
	upsample	2×		52×52
	route			
3×	conv	256	1×1	52×52
	conv	512	3×3	52×52
	conv	18	1×1	52×52
	detection			
	route			
	conv	128	1×1	52×52
	upsample	2×		104×104
	route			
3×	conv	128	1×1	104×104
	conv	256	3×3	104×104
	conv	18	1×1	104×104
	detection			

**Table 2:** The cyclist detection rate with different region proposal methods.

Region Proposal Method	Detection Rate (%)
ACF-PR	100%
ACF	69.22%

**Table 3:** Comparison with other popular detection methods.

Method	Code Type	AP (%)	Time (s)
ACF-RP-YOLO	MATLAB+C	82.69%	0.35
YOLOv3 [31]	C	69.00%	0.03
SSD [28]	PYTHON	57.42%	0.15
LDCF [5]	MATLAB	46.24%	1.5
ACF [40]	MATLAB	41.01%	0.18

**Table 4:** The consuming time and code type of each part of ACF-RP-YOLO.

Parts of ACF-PR-YOLO	Time (s)	Code Type
ACF-RP	0.18	MATLAB
YOLOv3	0.164	C
post-processing	0.003	MATLAB

## Abbreviations

The following abbreviations are used in this manuscript:

ACF	Aggregated channel feature
ACF-PR	Aggregated channel feature based region proposal method
YOLO	You only look once
TDCB	Tsinghua-Daimler cyclist benchmark
ADAS	Advanced driver assistance systems
VRU	Vulnerable road users
HOG	Histogram of oriented gradients
SVM	Support vector machine
DPM	Deformable part model
LDCF	Locally decorrelated channel feature
ROI	Region of interest
R-CNN	Region based convolutional network
Fast R-CNN	Fast region based convolutional network
Faster R-CNN	Faster region based convolutional network
Fused DNN	fused deep neural network
SSD	Single shot multibox detector
LUV	CIELUV color space
modelDs	Model height and width without padding
modelDsPad	Model height and width with padding
nNeg	max number of negative windows to sample
nAccNeg	max number of negative windows to accumulate
NMS	Nom-maximum suppression
VGG16	Visual geometry group network with 16 layers
AP	Average precision
GPU	Graphics processing unit
CPU	Central processing unit
